# Psychological–Emotional Aspect and Lifestyle in Professional Males Rugby Athletes

**DOI:** 10.3390/nu17020305

**Published:** 2025-01-16

**Authors:** Roberto Palazzo, Riccardo Bevilacqua, Marco Corsi, Edoardo Falconi, Sara Rosa, Laura Stefani

**Affiliations:** Sports Medicine Centre, University of Florence, 50121 Firenze, Italy; roberto.palazzo@unifi.it (R.P.); riccardo.bevilacqua@edu.unifi.it (R.B.); marco.corsi@unifi.it (M.C.); edoardo.falconi@unifi.it (E.F.); sara.rosa@unifi.it (S.R.)

**Keywords:** body composition, rugby players, MEDI-LITE, SF-36, BIA, IPAQ, mental health, physical activity, athletes

## Abstract

Background: Recent research emphasizes the importance of integrating psychological–emotional factors with nutrition and body composition in athletes. This study investigates the correlations between these aspects in 36 professional rugby players, aiming to identify relationships that could optimize sports performance and overall well-being. Methods: The study sample included 36 male athletes (mean age: 24.4 ± 2.1 years, weight: 86.5 ± 7.1 kg, and height: 181.8 ± 5.7 cm). Four assessment tools were used: the Short Form Health Survey Questionnaire (SF-36) to evaluate quality of life, the Mediterranean diet adherence (MEDI-LITE), the International Physical Activity Questionnaire (IPAQ), and body composition measurements (stadiometer and BIVA). Statistical analyses, including the Shapiro–Wilk test, Pearson, and Spearman correlations, were conducted to explore relationships between the variables. Results: The athletes’ mental and physical health was comparable to the general population (mean MCS = 50.5 ± 7.4, PCS = 49.6 ± 9.6). Mediterranean diet adherence was low (mean MEDI-LITE = 8.6 ± 2.6), while physical activity levels were high (mean IPAQ = 2560.5 ± 950.1). Body composition indicated overweight status (mean BMI = 26.15 ± 1.62 kg/m^2^, body fat = 22 ± 4.3%). Positive correlations were observed between mental health, diet adherence, and physical activity, with a stronger link between physical activity and body composition. Conclusions: This study confirms a relationship between psychological–emotional factors, nutrition, and body composition in athletes, suggesting an integrated approach to optimize performance. However, causality remains unconfirmed, and the small sample size limits generalizability. Future research should include larger, more diverse samples to expand these findings.

## 1. Introduction

In recent decades, numerous scientific studies explored the relationship between sports, nutrition, and hydration, highlighting how these factors significantly impact athletic performance and the physical well-being of athletes [1]. Specifically, nutrition has been recognized as a key element not only for maintaining health, but also for enhancing physical performance, particularly among elite athletes. Similarly, proper hydration has been shown to be essential in preventing fatigue and injuries, ensuring the efficient functioning of the physiological processes involved in athletic activity [2].

In addition to these traditionally studied aspects, there is growing interest in the psychological factor in sports performance [3,4], as mental well-being and psychological conditions are now recognized as crucial elements for achieving optimal performance. More recently, research has begun to explore the link between the gut and the brain, an innovative area that reveals how nutrition can directly influence not only physical health, but also psychological well-being. Preliminary studies suggest that a balanced diet that supports gut health may have positive effects on mental health, thereby indirectly enhancing athletic performance [5].

Despite these advances, the current literature still presents significant gaps regarding the analysis of the psychological aspect in elite athletes and how it integrates with variables such as body composition and dietary habits. This topic represents a promising yet underexplored field of study that could offer new perspectives on optimizing athlete performance and well-being through a more integrated approach that considers both body and mind in synergy.

The Mediterranean diet (MD), recognized as one of the healthiest dietary patterns worldwide, emphasizes the consumption of fruits, vegetables, whole grains, olive oil, nuts, and fish, while limiting red meat and processed foods. This diet is known not only for its potential to improve physical health outcomes, but also for its association with better psychological well-being and quality of life. However, there is limited research on how adherence to the MD affects professional athletes, particularly in terms of its interaction with psychological factors and overall performance. Understanding this relationship could provide a valuable framework for improving the health and performance of athletes through tailored dietary interventions.

Quality of life (QoL) is another critical component when evaluating the well-being of athletes. Defined as an individual’s overall perception of their physical and mental health, QoL encompasses multiple domains, including physical functioning, emotional stability, and social engagement. In elite sports, where physical and mental demands are high, understanding how lifestyle factors and dietary habits influence QoL can offer new insights into supporting athletes in achieving a balanced and sustainable career.

Furthermore, the sport of rugby, characterized by its intense physical demands and team-oriented nature, offers a unique context for studying these variables. Rugby players face a combination of strength, endurance, and tactical challenges that require optimal physical fitness and mental resilience. The sport’s specific characteristics—such as high-intensity collisions, strategic teamwork, and sustained physical exertion—make it an ideal setting to examine how nutrition, hydration, and psychological factors interact to influence both performance and well-being.

This study focuses on the potential correlation between lifestyle variables and emotional factors in professional rugby players. By analyzing adherence to the Mediterranean diet, levels of physical activity, and quality of life, this research aims to contribute to the understanding of how these interconnected variables shape the overall health and performance of athletes in a high-demand sport such as rugby.

## 2. Materials and Methods

### 2.1. Study Population

The study was conducted in a group of 36 male professional rugby athletes, with an average weight of 86.5 ± 7.1 kg, an average height of 181.8 ± 5.7 cm, an average age of 24.4 ± 2.1 years, an average body mass index (BMI) of 26.1 ± 1.6 kg/m^2^, and an average of 7 years of sports experience.

Each participant provided written informed consent for the data treatment. The administration of the questionnaires and the collection of anthropometric data took place during a routine training period, ensuring that the responses accurately reflected the athletes’ typical physical and psychological state. Among the questionnaires used, the Short Form Health Survey Questionnaire (SF-36), International Physical Activity Questionnaire (IPAQ), and MEDI-LITE were included. Considering this is the team’s first exploration and not a seasonal evaluation, all interviews were conducted at a single point during the initial assessment.

### 2.2. SF-36 Questionnaire

The SF-36 questionnaire [6] was used to assess health-related quality of life, divided into eight areas: physical functioning, role limitations due to physical and emotional problems, bodily pain, general health, vitality, social functioning, and mental health. From these dimensions, two summary indices can be calculated: the physical component summary (PCS) and the mental component summary (MCS).

To combine the different scales that make up the SF-36 into two summary indices, each scale is standardized. This is accomplished by subtracting the average score of the reference population from the subject’s score and dividing the result by the standard deviation of the population. This process ensures that the scores of the different scales are comparable. The formula used is as follows:

Z scale = (P scale − μ scale)/σ scale

where:

- P scale is the raw score of the scale for the individual.
- μ scale is the mean score of the scale in the reference population.
- σ scale is the standard deviation of the scale in the reference population.

After standardization, each scale is weighted by a specific coefficient that reflects the contribution of that scale to the physical or mental health score. The 8 scales do not have the same weight in the two indices (PCS and MCS). Some scales have a greater impact on physical health (such as physical functioning or bodily pain), while others primarily affect mental health (such as mental health or vitality).

- For the PCS: The scales of physical functioning (0.42), physical role limitations (0.35), and bodily pain (0.32) have a higher weight. Emotional role limitations (−0.19), mental health (−0.22), social functioning (−0.01), general health (0.25), and vitality (0.3) have a lower weight.
- For the MCS: The scales of mental health (0.49), vitality (0.24), and emotional role limitations (0.43) have a higher weight. Physical functioning (−0.23), physical role limitations (−0.12), bodily pain (−0.1), social functioning (0.27), general health (−0.02), and vitality (0.24) have a lower weight.

Once standardized and weighted, the scales are summed to obtain two overall indices: the physical component summary (PCS) and the mental component summary (MCS). The general formulas to calculate the summary indices are:

PCS = Σ (Z scale × W scale)

MCS = Σ (Z scale × W scale)

where:

Z scale is the standardized score for each scale.

W scale is the weight assigned to each scale for the MCS.

The PCS and MCS indices are interpreted in relation to a reference population with an average score of 50 and a standard deviation of 10. Therefore, a score above 50 indicates physical or mental health above the population average, while a score below 50 indicates health below the average.

These two indices provide a concise overview of an individual’s physical and mental health, allowing for the identification of potential issues or strengths in these areas.

### 2.3. IPAQ

The IPAQ was administered to assess the physical activity level of the athletes [7]. This tool classifies individuals based on the amount of physical activity performed, allowing for the distinction between light, moderate, and vigorous activity levels. During the interview, participants were asked to describe in detail the number, type, and duration of their training sessions. The data were then divided by 7 to obtain the average daily training time, which, when added to other daily activities performed by each individual, enabled the precise calculation of the exercise energy expenditure (EEE), measured in METs.

In more detail, each type of activity is assigned a MET value (an energy cost unit for the activity):

- Vigorous activity: 8.0 MET.
- Moderate activity: 4.0 MET.
- Walking: 2.5 MET for slow pace, 3.0 MET for moderate pace, and 3.3 MET for brisk pace.

The IPAQ score is expressed in MET-minutes per week, calculated by multiplying the specific MET value by the number of minutes per day and the number of days the activity was performed. For each activity level (vigorous, moderate, and walking), a MET-minutes score is obtained, and these are summed to obtain the total IPAQ score. After calculating the total score, the individual is classified into one of the following three categories:

- Inactive: 699 or fewer METs per week (cells marked in red).
- Sufficiently active: between 700 and 2519 METs per week (cells marked in yellow).
- Active or very active: 2520 or more METs per week (cells marked in green).

### 2.4. MEDI-LITE Questionnaire

The MEDI-LITE was used to assess adherence to the Mediterranean diet guidelines, considered as a protective factor for cardiovascular health and general well-being [8,9]. The questionnaire examines dietary habits on a daily and weekly basis, providing parameters for various components of the diet. Each component is scored from 0 to 2 based on the frequency of consumption. The scores for each food group are summed to obtain a total score ranging from 0 to 18:

- Low score (≤8): indicates poor adherence to the Mediterranean diet.
- Moderate score (9–11): indicates moderate adherence to the Mediterranean diet.
- High score (≥12): indicates high adherence to the Mediterranean diet.

### 2.5. Body Composition Analysis

Following the administration of the questionnaires, each athlete underwent measurements of weight and height, which were taken using a stadiometer. During these measurements and the bioelectrical impedance analysis (BIA), participants were required to wear only underwear.

Subsequently, a bioimpedance examination was conducted using the Akern BIA 101 BIVA^®^ PRO device [10,11,12]. All tests were performed around 7 p.m., prior to training, at the playing field with an external temperature of 8 °C and an internal temperature of 22 °C. The tools used for the measurements included a scale, a stadiometer, and the Akern bioimpedance device. No dietary restrictions were imposed on the participants. They followed non-specific dietary guidelines or restrictions. The protocol for anthropometric evaluation was performed using BIA as follows: Fasting for the previous 24 h, avoiding excessive consumption of foods rich in caffeine (such as chocolate, dark tea, and coffee) for two days before the exam. The day before the exam, participants were instructed not to engage in intense physical activity or use the sauna. This approach was chosen to reflect the athletes’ actual physical conditions during a typical training day, and to highlight any behaviors and habits that might not be ideal for maximizing athletic performance. All participants were in good health at the time of the examination, and none had pacemakers or other electronic devices implanted. It should be noted, however, that the results might have been slightly influenced by the fact that one of the participants recently recovered from a prolonged illness.

Each subject was placed one by one on a reclining table, lying in a supine position with the body completely relaxed. The arms were positioned approximately 30° away from the trunk, and the legs were spread at about 45°, to prevent contact between the limbs, which could interfere with the electrical current flow and result in inaccurate measurements. For the same reason, all jewellery and metallic objects were removed. Before applying the electrodes, the skin area to be measured was cleaned with an alcohol-soaked cloth and, if necessary, shaved.

The first electrode was placed on the dorsal side of the right foot, over the first metatarsal joint. The second electrode was placed on the right heel, at the back of the foot.

The test was only conducted five minutes after the athlete assumed the correct position, allowing sufficient time for the physiological and more uniform redistribution of body fluids due to gravity.

Participants were instructed to remain still, relaxed, and silent throughout the measurement process.

### 2.6. Statistical Analysis

The data obtained were tabulated, and the mean and standard deviation for each variable derived from the questionnaires, anthropometric analysis, and BIA were calculated. Additionally, weight and height were categorized into ranges (5 kg for weight and 5 cm for height) to provide a quicker overview of the data relative to the reference population. For the same purpose, the age of the participants was represented using a histogram.

For each variable of interest, a normality test (Shapiro–Wilk test) was performed with an alpha level of 0.05 (95% significance level) to assess whether the data distribution followed a normal (Gaussian) distribution.

Finally, the degree of linear correlation between each pair of variables was analyzed using Pearson’s linear correlation method when both variables met the assumption of normality, or Spearman’s linear correlation method when one or both variables did not follow a normal distribution. For the latter method, it was also necessary to perform a rank calculation, which involves assigning an ordinal number (or rank) to each data point based on its relative position within the dataset, for any variable with a non-normal distribution.

The results obtained from the two statistical methods were tabulated. Correlations with values between −0.7 and −0.5, and between 0.5 and 0.7 (indicating moderate positive or negative trends), were highlighted in yellow. More significant correlations, with values between −1 and −0.7, or between 0.7 and 1 (indicating strong positive or negative correlations), were highlighted in red.

## 3. Results

All data are expressed as mean ± SD. All the data were within the normal range, with the exception of the MEDILite score, which was below the normal range (>13). The average data fell within a normal range; in fact, the IPAQ indicated that they were active individuals (>700 METs), and the SF-36 showed values all close to the population averages.

All the athletes of the rugby team were enrolled, with the exception of subject no. 14 due to his recent recovery from a prolonged injury.

### 3.1. SF-36 Questionnaire

The data obtained from the SF-36 questionnaire reveal that all three areas related to physical health exhibit the following mean values:

- Physical functioning: 98.19 ± 4.95.
- Role limitations due to physical health: 84.72 ± 25.55.
- Physical pain: 79.31 ± 19.84.

The three areas related to psychological and emotional health show the following mean values:

- Role limitations due to emotional problems: 70.39 ± 39.67.
- Mental health: 69.22 ± 17.88.
- Social functioning: 72.22 ± 21.36.

The two areas related to general health show the following mean values:

- General health: 72.08 ± 15.32.
- Vitality: 60.28 ± 14.97.

Finally, the change in health over time shows the following mean value:

- Health change: 56.25 ± 25.62.

It is also noticeable that the values related to psychological–emotional health and health change exhibit greater variability compared to those related to physical health and general health:

- Mean standard deviation of the three areas related to physical health: 16.78.
- Mean standard deviation of the three areas related to psychological–emotional health: 26.3.
- Mean standard deviation of the two areas related to general health: 15.15.
- Standard deviation of the health change variable: 25.62.

The physical component summary (PCS) and the mental component summary (MCS) show values that are perfectly in line with those of the general population (standardized value of 50):

- Physical component summary: 49.55 ± 9.16.
- Mental component summary: 50.48 ± 7.05.

These results suggest that, on average, the athletes’ perceived physical and mental health are consistent with the general population, reflecting a balanced state of health in these domains. However, the higher variability in the psychological–emotional and health change areas suggests more individual differences within the group in these aspects of well-being.

### 3.2. IPAQ

The administration of the IPAQ questionnaire reveals that the average level of physical activity for the athletes in the study is 3114.1 ± 1835.51 MET. A more detailed analysis shows that 22 out of the 36 athletes (61%) have a score of 2520 MET or higher, categorizing them as active or very active. Thirteen athletes, representing a significant proportion (36%), have scores between 700 and 2519 MET, thus falling into the sufficiently active category. Finally, only one athlete (3%), who has been injured, shows a score below 700 MET, placing them in the inactive category.

These results highlight that the majority of the athletes are highly active, with a small percentage falling into the less active categories, primarily due to injury or other factors affecting their activity level.

### 3.3. MEDI-LITE

The data obtained from the MEDI-LITE questionnaire show an average score of 8.61 ± 2.58 points, which places the athletes just above the threshold between the categories of “poor adherence to the Mediterranean diet” and “moderate adherence to the Mediterranean diet”. A closer look reveals that 15 athletes (42%) fall into the poor adherence category, 16 athletes (44%) are classified in the moderate adherence category, and 5 athletes belong to the high adherence to the Mediterranean diet category.

These results suggest that the majority of athletes display moderate adherence to the Mediterranean diet, with a notable proportion showing poor adherence, and a smaller group exhibiting high adherence. This distribution indicates some variability in dietary habits among the athletes, with room for improvement in aligning with the Mediterranean diet.

### 3.4. Anthropometric Measurements and Bioimpedance

The measurements obtained using the stadiometer show an average weight of 86.5 ± 7.07 kg and an average height of 181.83 ± 5.66 cm. This results in an average body mass index (BMI) of 26.15 ± 1.62 kg/m^2^, which is above the 25 kg/m^2^ threshold for the normal weight category. Analyzing the data subject by subject, only 9 athletes (25%) are within the normal weight range, while 27 athletes (75%) are classified as overweight. There are no athletes classified as underweight. The data from the BIA analysis are presented in Table 1.

A Spearman correlation analysis was conducted, highlighting several significant correlations. A weak positive correlation emerged between ISF and physical functioning (0.54) and between ISF and role physical limitations (0.61). Additionally, a positive correlation was observed between ISM and emotional role limitations (0.60), between IPAQ and mental health (0.54), and between MEDILITE and mental health (0.58).

**Figure 1:**
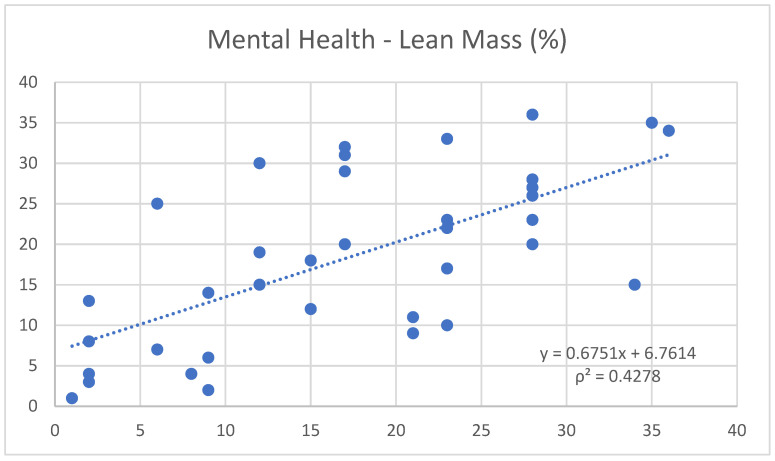
Correlation between the variables “mental health” and “lean mass”.

**Figure 2:**
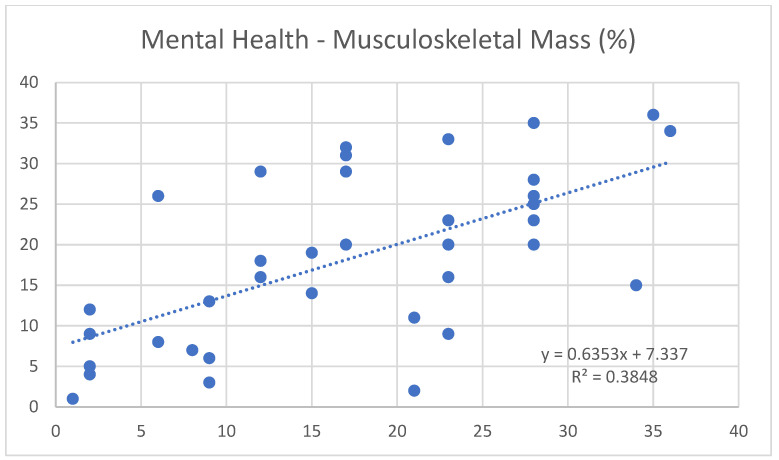
Correlation between the variables “mental health” and “musculoskeletal mass”.

**Figure 3:**
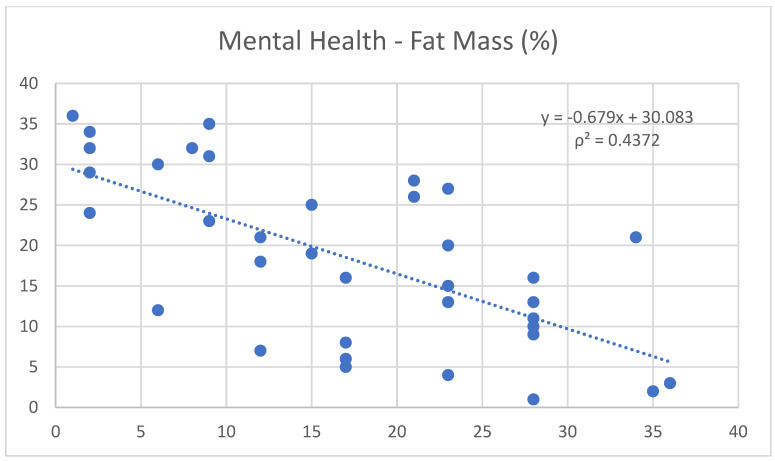
Correlation between the variables “mental health” and “fat mass”.

Moreover, noteworthy correlations were found between mental health and free fat mass (0.65) (Figure 1), and between mental health and Janssen SMM (0.62) (Figure 2). Conversely, it shows a negative trend with fat mass (−0.66) (Figure 3).

This shows how mental health can be strongly related to body composition, even in highly trained individuals. In particular, adherence to the Mediterranean diet can make a significant difference. On the other hand, a countertrend and lower impact are evident when other variables, such as the IPAQ score, are considered.

Fat mass was negatively correlated with IPAQ (−0.50) and MEDILITE (−0.67), while free fat mass exhibited a positive correlation with MEDILITE (0.66).

Strong correlations were identified, with ISF and physical pain showing a correlation of 0.76 (>0.7), and IPAQ and fat mass showing a correlation of −0.71 (<−0.7).

The full statistical analysis, along with all related data, are available in the Supplementary File and may be requested from the corresponding author.

**Figure 4:**
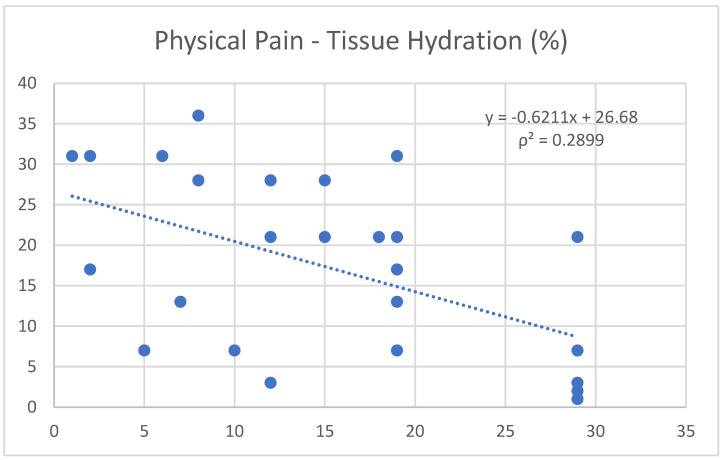
Correlation between the variables “physical pain” and “tissue hydration”.

**Figure 5:**
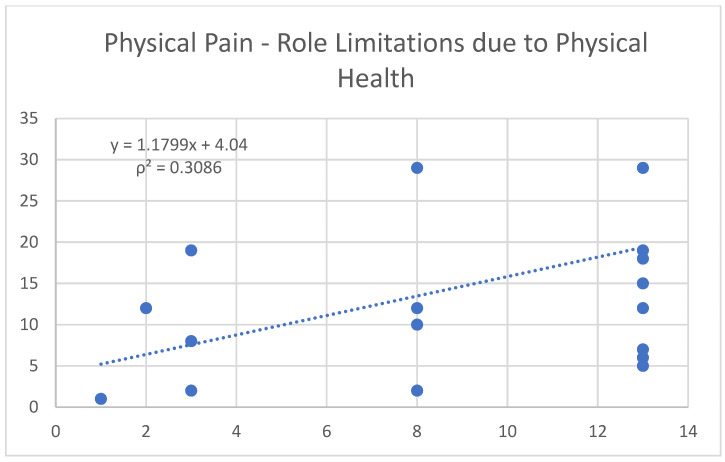
Correlation between the variables “physical pain” and “role limitations” due to physical health.

Physical pain not only shows a moderate negative correlation with tissue hydration (−0.54) (Figure 4), but also exhibits a positive trend with role limitations due to physical health (0.56) (Figure 5).

All values obtained from bioelectrical impedance analysis fall within the normal ranges for individuals of the same age and sex [13].

## 4. Discussion

Psychological and emotional status in elite athletes has been widely studied; however, the potential correlation with other aspects of lifestyle, including body composition, has not been thoroughly investigated. The data obtained support the role of adherence to the Mediterranean diet, as well as adequate body composition, particularly in terms of hydration status. The results from the administration of the SF-36 questionnaire interestingly show that the psychological–emotional and physical health levels of the professional athletes analyzed are in line with those of the general population. From the retrospective analysis conducted through the questionnaire, it appears that the health status during the four weeks preceding the completion of the questionnaire also fall within the population’s average.

What is most interesting, however, is the association found between the aforementioned variables and the psychological–emotional aspects of the professional athletes. Specifically, both adherence to the Mediterranean diet and physical activity levels were positively associated with improvements in the athletes’ mental health. However, they do not seem to have the same effect. Adherence to the Mediterranean diet appears to have a greater impact, particularly in the presence of high levels of physical activity.

This could be explained by the fact that a proper intake of high-quality essential nutrients can positively influence neurotransmitter levels and neuronal plasticity, leading to better cognitive function, increased emotional resilience, and reduced depressive symptoms [14,15,16]. At the same time, the Mediterranean diet is known to reduce oxidative stress levels in the body due to its high antioxidant content. This can help decrease chronic inflammation, mitochondrial dysfunction, and impaired neurogenesis, ultimately protecting against neuronal damage caused by free radicals and contributing to the positive effects on mental health [17].

In parallel, regular physical exercise is known to stimulate endorphin production, which, along with improvements in self-perception and physical condition, helps to reduce stress levels and enhance mood [18,19]. This statement is supported by the positive correlation found in the study between mental health and free fat mass (0.65) and Janssen SMM (0.62), as well as the negative correlation with fat mass (−0.66).

Another significant finding is the negative correlation between physical pain and tissue hydration. Dehydration can negatively affect muscle recovery capacity, increase pain perception, and especially lower mental performance and concentration, thereby exposing the athlete to a greater risk of injury [2,20]. Physical pain is also positively correlated with the limitations in the athlete’s ability to perform daily activities, including sports. This was evidenced by a strong correlation between ISF and physical pain (0.76), and a moderate negative correlation between physical pain and tissue hydration (−0.54). Physical pain also exhibited a positive correlation with role limitations in physical health (0.56).

Finally, it is interesting to note that age does not appear to correlate with any of the other considered variables. This could be explained by the relative homogeneity of the sample in this regard, which reduces the variability of this parameter.

## 5. Conclusions

The study results clearly highlight that the mental health of professional rugby athletes is significantly influenced by both lifestyle and body composition. Specifically, a positive correlation emerges between psychological well-being and adherence to the Mediterranean diet, as well as with levels of physical activity. These findings align with numerous scientific studies that connect the quality of diet and physical activity not only to physical health, but also to the psychological–emotional domain.

The study emphasizes that body composition data, which are more tangible and easily measurable, and often used as the sole reference in overall athlete evaluation, require a more integrated approach that also takes psychological dimensions into account for maximizing sports performance and preventing injuries.

Further investigation into these aspects in future research could provide additional tools to optimize athlete health through integrated approaches that encompass diet, physical exercise, and psychological management.

## Figures and Tables

**Table 1:** Averages and SD of BIA. The average values derived from the bioelectrical impedance analysis are within the normal range, except for the fat mass, which is at the upper limits.

BIA Averages and SD	
Phase angle (Pha)	7.13 ± 0.4°
Total body water (TBW)	57.33 ± 3.25%
Extracellular water (ECW)	41.07 ± 1.4 8%
Intracellular water (ICW)	58.93 ± 1.48%
Tissue hydratation (TBW/FFM)	73.18 ± 0.24
Lean body mass (FFM)	78.34 ± 4.34%
Fat mass (FM)	21.66 ± 4.34%
Cell mass (BCM)	58.86 ± 1.68%
Total muscle mass (MM)	55.94 ± 3.7%
Skeletal muscle mass (SMM) (Janssen method)	41.29 ± 3.39%
Appendicular skeletal muscle mass (ASMM)	27.26 ± 4.7 kg
Basal metabolic rate (BMR)	1904.28 ± 93.41 kcal
Total daily energy expenditure (TDEE)	3237.28 ± 158.8 kcal

## Data Availability

The original contributions presented in the study are included in the article, further inquiries can be directed to the corresponding author.

## Supplementary Materials

The following supporting information can be downloaded at: https://www.mdpi.com/article/10.3390/nu17020305/s1, Table S1: Pearson Correlation Coefficients; Table S2: Spearman Correlation Coefficients.
