# Supplementary material for: Psychological–Emotional Aspect and Lifestyle in Professional Males Rugby Athletes
Source: Nutrients. 2025 Jan 16;17(2):305. doi: 10.3390/nu17020305 (PMC11767787; doi:10.3390/nu17020305)
Supplement: Supplementary file 1 [file nutrients-17-00305-s001.zip › nutrients-3412498-supplementary.pdf]

nutrients

MDPI

Table S1: Pearson Correlation Coefficients.

Pearson Correlation Coefficients	General Health	MEDI-LITE	Fat-Free Mass (FFM)	Fat Mass (FM)	SMM Janssen
General Health	1.00	0.06	0.06	-0.06	0.03
MEDI-LITE	0.06	1.00	0.63	-0.63	0.66
Fat-Free Mass (FFM)	0.06	0.63	1.00	-1.00	0.99
Fat Mass (FM)	-0.06	-0.63	-1.00	1.00	-0.99
SMM Janssen	0.03	0.66	0.99	-0.99	1.00

Table S2: Spearman Correlation Coefficients.

	Physical Functioning	Role Limitations due to Physical Health	Physical Pain	Role Limitations due to Emotional Health	Mental Health	Social Functioning	General Health	Vitality	Health Change	PCS	MCS	IPAQ
Physical Functioning	1.00	0.14	0.25	-0.13	-0.07	0.21	0.27	0.25	0.20	0.54	-0.23	0.10
Role Limitations due to Physical Health	0.14	1.00	0.56	0.33	-0.06	0.22	0.17	0.09	0.16	0.61	-0.05	-0.24
Physical Pain	0.25	0.56	1.00	-0.04	0.10	0.34	0.24	0.36	-0.02	0.76	-0.01	-0.06
Role Limitations due to Emotional Health	-0.13	0.33	-0.04	1.00	-0.05	0.20	0.14	0.20	0.00	0.00	0.60	-0.12
Mental Health	-0.07	-0.06	0.10	-0.05	1.00	-0.11	0.15	-0.14	-0.14	-0.20	0.46	0.54
Social Functioning	0.21	0.22	0.34	0.20	-0.11	1.00	0.22	0.48	0.16	0.37	0.40	-0.26
General Health	0.27	0.17	0.24	0.14	0.15	0.22	1.00	0.37	-0.25	0.50	0.29	0.14
Vitality	0.25	0.09	0.36	0.20	-0.14	0.48	0.37	1.00	-0.08	0.44	0.36	0.01
Health Change	0.20	0.16	-0.02	0.00	-0.14	0.16	-0.25	-0.08	1.00	0.05	-0.16	0.08
PCS	0.54	0.61	0.76	0.00	-0.20	0.37	0.50	0.44	0.05	1.00	-0.16	-0.19
MCS	-0.23	-0.05	-0.01	0.60	0.46	0.40	0.29	0.36	-0.16	-0.16	1.00	0.19
IPAQ	0.10	-0.24	-0.06	-0.12	0.54	-0.26	0.14	0.01	0.08	-0.19	0.19	1.00
MEDI-LITE	-0.37	-0.31	-0.20	-0.04	0.58	-0.20	0.08	-0.12	-0.21	-0.37	0.34	0.32
Age (years)	0.09	0.05	-0.14	0.20	-0.11	0.07	-0.09	-0.02	0.14	-0.02	0.14	-0.28
BMI (kg/m^2)	0.04	0.16	-0.06	0.29	-0.45	0.17	-0.22	0.09	-0.14	0.03	-0.07	-0.71
Tissue Hydration	-0.34	-0.23	-0.54	0.17	-0.08	-0.12	-0.13	-0.30	-0.03	-0.43	0.12	-0.18
Phase Angle (Pha)	0.16	0.18	0.15	0.01	0.26	0.05	0.04	0.07	-0.03	-0.02	0.14	0.06
Fat-Free Mass (FFM)	-0.12	-0.13	-0.20	-0.09	0.65	-0.26	-0.02	-0.33	0.08	-0.38	0.21	0.49
Fat Mass (FM)	0.11	0.12	0.19	0.08	-0.66	0.26	0.01	0.31	-0.07	0.37	-0.22	-0.50
SMM Janssen	-0.11	-0.12	-0.22	-0.05	0.62	-0.23	-0.02	-0.31	0.07	-0.38	0.23	0.47

MEDI-LITE	Age (years)	BMI (kg/m^2)	Tissue Hydration	Phase Angle (Pha)	Fat Mass (FM)	SMM Janssen
-0.37	0.09	0.04	-0.34	0.16	0.11	-0.11
-0.31	0.05	0.16	-0.23	0.18	0.12	-0.12
-0.20	-0.14	-0.06	-0.54	0.15	0.19	-0.22
-0.04	0.20	0.29	0.17	0.01	0.08	-0.05
0.58	-0.11	-0.45	-0.08	0.26	-0.66	0.62
-0.20	0.07	0.17	-0.12	0.05	0.26	-0.23
0.08	-0.09	-0.22	-0.13	0.04	0.01	-0.02
-0.12	-0.02	0.09	-0.30	0.07	0.31	-0.31
-0.21	0.14	-0.14	-0.03	-0.03	-0.07	0.07
-0.37	-0.02	0.03	-0.43	-0.02	0.37	-0.38

0.34	0.14	-0.07	0.12	0.14	-0.22	0.23
0.32	-0.28	-0.71	-0.18	0.06	-0.50	0.47
1.00	-0.24	-0.46	0.19	0.09	-0.67	0.67
-0.24	1.00	0.40	0.00	0.17	0.19	-0.20
-0.46	0.40	1.00	0.14	0.07	0.56	-0.55
0.19	0.00	0.14	1.00	-0.50	-0.23	0.25
0.09	0.17	0.07	-0.50	1.00	-0.29	0.26
0.66	-0.19	-0.56	0.23	0.28	-1.00	0.99
-0.67	0.19	0.56	-0.23	-0.29	1.00	-0.99
0.67	-0.20	-0.55	0.25	0.26	-0.99	1.00

Fat-Free Mass (FFM)	Fat Mass (FM)	SMM Janssen
-0.12	0.11	-0.11
-0.13	0.12	-0.12
-0.20	0.19	-0.22
-0.09	0.08	-0.05
0.65	-0.66	0.62
-0.26	0.26	-0.23
-0.02	0.01	-0.02
-0.33	0.31	-0.31
0.08	-0.07	0.07
-0.38	0.37	-0.38
0.21	-0.22	0.23
0.49	-0.50	0.47
0.66	-0.67	0.67
-0.19	0.19	-0.20
-0.56	0.56	-0.55
0.23	-0.23	0.25
0.28	-0.29	0.26
1.00	-1.00	0.99
-1.00	1.00	-0.99
0.99	-0.99	1.00
